# Uptake and Distribution of Fenoxanil-Loaded Mesoporous Silica Nanoparticles in Rice Plants

**DOI:** 10.3390/ijms19102854

**Published:** 2018-09-20

**Authors:** Feng Zhu, Xingang Liu, Lidong Cao, Chong Cao, Fengmin Li, Caijun Chen, Chunli Xu, Qiliang Huang, Fengpei Du

**Affiliations:** 1 State Key Laboratory for Biology of Plant Diseases and Insect Pests, Institute of Plant Protection, Chinese Academy of Agricultural Sciences, 2 Yuanmingyuan West Road, Haidian District, Beijing 100193, China; gzzbszf@163.com (F.Z.); liuxingang@caas.cn (X.L.); caolidong@caas.cn (L.C.); ccao@ippcaas.cn (C.C.); fmli@ippcaas.cn (F.L.); springxcl2013@126.com (C.X.); 2 Institute of Plant Protection, Guizhou Academy of Agricultural Sciences, Huaxi District, Guiyang 550006, China; chencj1123@126.com; 3 Department of Applied Chemistry, College of Science, China Agricultural University, 2 Yuanmingyuan West Road, Haidian District, Beijing 100193, China

**Keywords:** mesoporous silica nanoparticles, Fenoxanil, uptake, distribution, rice

## Abstract

Mesoporous silica nanoparticles (MSNs) can be used as carriers to deliver pesticides into plants, which is considered to be one method of improving the efficacy of pesticide usage in agricultural production. In the present work, MSNs with an average diameter of 258.1 nm were synthesized and loaded with Fenoxanil. The structure of the nanocarriers was observed by scanning electron microscopy. The loading content of Fenoxanil-loaded MSNs was investigated. After rice plants in a hydroponic system were treated with loaded MSNs, the concentrations of Fenoxanil in different samples were determined using high-performance liquid chromatography–tandem mass spectrometry. The results suggested that rice plants can absorb MSNs from water through their roots, and the dosage has almost no effect on the distribution of Fenoxanil in rice plants. The application of pesticide-loaded nanoparticles in a hydroponic system poses a low risk of Fenoxanil accumulation in rice.

## 1. Introduction

The application of pesticides can be affected by many factors. The main factors include the type and properties of the plants on which the pesticides are used in the field. The uptake and translocation behaviors of pesticides can affect their effectiveness in plants [1]. Pesticides can be classified as systemic or non-systemic, according to their mode of action. Systemic pesticides can be absorbed by plants and transported from the site of application to other parts of the plant, thereby improving the plant’s resistance to disease. Once absorbed, systemic pesticides cannot be affected by environmental factors, such as rainfall and climate. They protect the roots from soil insects and soil-borne diseases, while also preventing pests and disease from spreading to the stems and leaves. On the contrary, non-systemic pesticides cannot penetrate into plants when sprayed on their leaves. As they only remain on the surface of plants, they cannot prevent insects from attacking new leaves or the spread of diseases in plant tissues. Different pesticides have different absorption and transmission properties in plants. Previous studies have shown that chlorantraniliprole can be detected in rice stems and leaves after addition to hydroponic culture solution, but it cannot be detected in rice roots after use of the spraying method [2]. The systemic fungicide flumorph shows strong conduction and redistribution to the apical and stratified layers in cucumber but has no ability to transport to the roots or between leaves [3]. Although acetoxazole can be transmitted to the substrate in tomato plants, this effect is relatively weak [4]. However, the penflufen can be conducted between the leaves of the plant, with both upward and downward bidirectional conduction characteristics [5]. In general, all pesticides show distinctive absorption and conduction properties in plants. Also, the efficacy of pesticides depends on various factors, such as the plant species, method of application, and period of growth. Therefore, improving the transportation performance of pesticides in plants is an important objective in improving the utilization of pesticides.

Since the first report of an ordered mesoporous material, named MCM-41 by Kresge et al. in 1992, the study of mesoporous silica nanoparticles (MSNs) has received extensive attention [6]. MSNs have good advantages, such as low cost, good environmental compatibility, large specific surface area, adjustable pore size, and high loading capacity. Controlled release of an active compound can be achieved after surface modification, and MSNs are widely used in therapeutics, drug delivery, and diagnostics [7,8,9,10,11,12]. In recent years, more and more MSNs have been used as pesticide carriers. When pyocyanin was loaded into MSNs to prepare a sustained-release system, the degradation of unstable pyocyanin could be effectively prevented [13]. The application of avermectin in field production has been restricted by time, due to its poor solubility and easy photodegradation. Avermectin-loaded silica nanoparticles demonstrated a remarkable performance, with greatly improved photostability, water solubility, and bioavailability of avermectin [14,15]. The cytotoxicity and antibacterial activity of tebuconazole could be improved when MSNs were used as carriers [16]. A sustained antibacterial effect was achieved for volatile essential oils when they were loaded into MSNs [17]. Wanyika et al. loaded MSNs with chlorantraniliprole, and the prepared pesticide-loaded nanoparticles showed a better sustained-release effect [18]. Owing to their good performance, MSNs have been used as vehicles to deliver DNA and chemicals into plants [19]. Mou et al. employed functionalized MSNs to develop an MSN-mediated plant transient gene expression system that carried exogenous DNA into the roots of *Arabidopsis thaliana* [20]. Zhao et al. conducted a study on the absorption and transport of pesticide-loaded MSNs in cucumbers, and the results indicated that MSNs can be absorbed and transmitted in cucumber after having been applied to the leaves [21,22].

**Figure 1:**
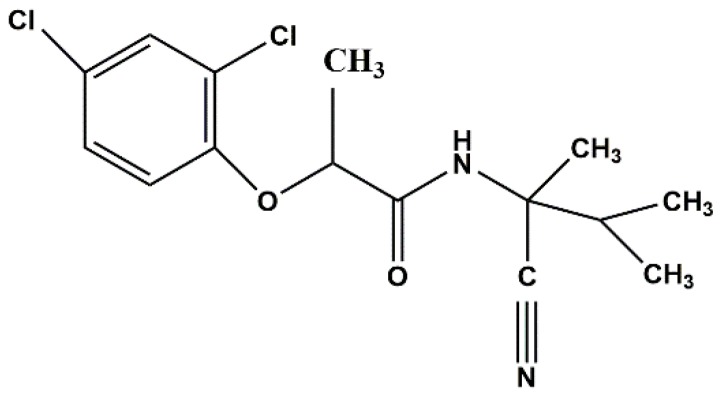
Structure of Fenoxanil.

Fenoxanil (CAS No. 115852-48-7, Figure 1) is a new systemic and protective fungicide with residual effects, belonging to the propionamide chemical class. Fenoxanil is a melanin biosynthesis inhibitor used to control rice blast caused by the fungus *Pyricularia oryzae* [23]. Some researchers have studied Fenoxanil residues [24] and have found that they may exert potentially adverse effects on human health and the environment.

Rice is grown in many countries and feeds billions of people around the world. However, it is quite susceptible to diseases, of which the most destructive is rice blast, caused by the fungi *P. oryzae*. It is therefore necessary to prevent rice from fungal diseases that lead to a loss of production, especially rice blast [25,26]. Various pesticides are applied to control rice diseases, and good control can be attained. However, adverse effects on humans and the environment may be caused by the low utilization efficiency of pesticide application. Adoption of MSNs as carriers could be a promising solution to some of the limitations modern pesticides are facing.

In the present work, MSNs with an average diameter of 258.1 nm were synthesized to obtain Fenoxanil-loaded MSNs (Fen@MSNs). Fen@MSNs were then applied to rice plants in order to characterize their uptake and distribution behaviors. Fenoxanil was chosen as the template pesticide, since it is widely used to control rice blast. After treatment of the rice plants with Fen@MSNs, the concentrations of Fenoxanil in different plant parts and in environmental samples were determined over a period of 10 days. In addition, the final residue level of Fenoxanil in rice was measured to evaluate the food safety of Fen@MSNs in rice.

## 2. Results and Discussion

### 2.1. Preparation and Characterization of MSNs and Fen@MSNs

**Figure 2:**
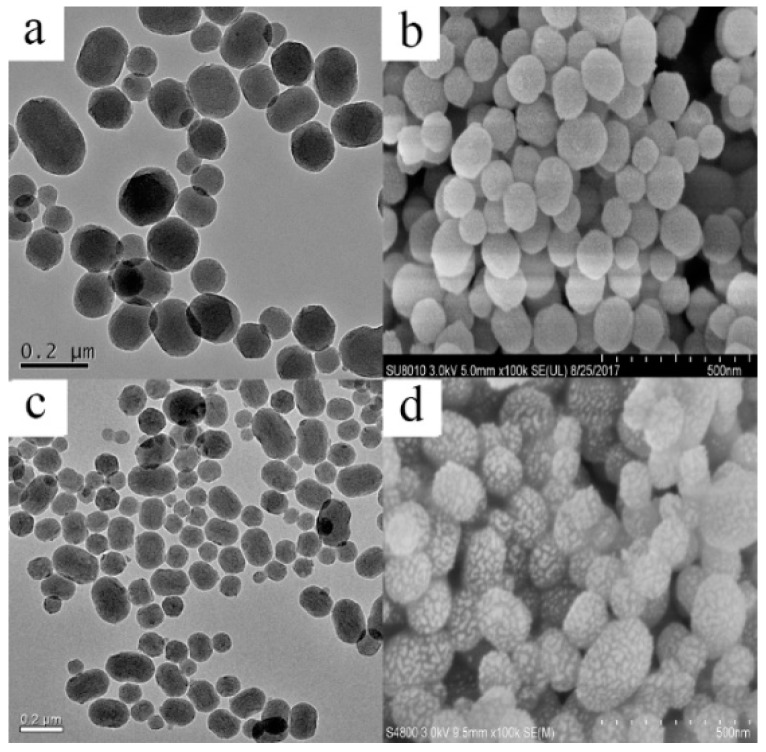
Scanning electron microscope (SEM) images: (a) mesoporous silica nanoparticles (MSNs), scale bar = 0.2 μm (c) Fenoxanil-loaded MSNs (Fen@MSNs), scale bar = 500 nm; transmission electron microscope (TEM) images: (b) MSNs, scale bar = 0.2 μm; (d) Fen@MSNs, scale bar = 500 nm.

**Figure 3:**
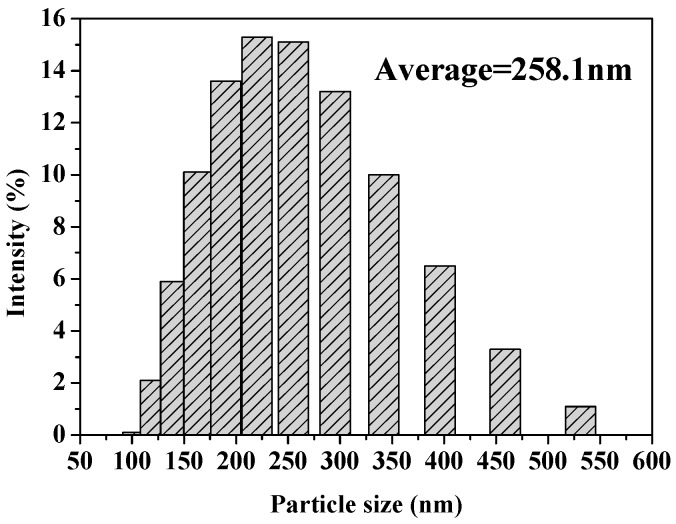
Histogram of the particle size distribution of MSNs.

**Figure 4:**
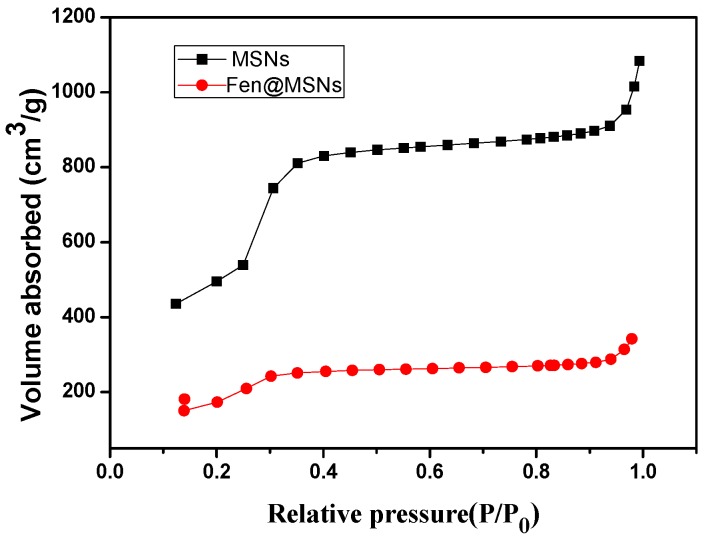
Nitrogen adsorption–desorption isotherms of MSNs and Fen@MSNs.

**Figure 5:**
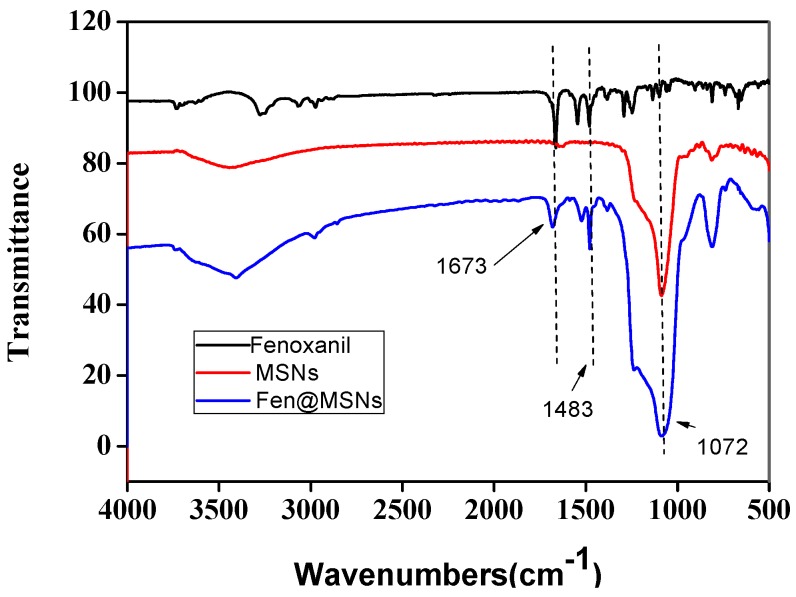
Fourier transform infrared (FTIR) spectra of Fenoxanil, MSNs, and Fen@MSNs.

In this study, the liquid crystal templating method was used to prepare MSNs, with tetraethyl orthosilicate (TEOS) as the silica precursor and cetyltrimethylammonium bromide (CTAB) as the surfactant in basic conditions. Pesticides were loaded into MSNs by simple immersion in a concentrated methanol solution of Fenoxanil. Because the uptake of MSNs can be affected by their size [27], the morphology of the MSNs and Fen@MSNs was characterized using a scanning electron microscope (SEM) and a transmission electron microscope (TEM). SEM images showed that there was no obvious difference between the MSNs and Fen@MSNs (Figure 2). Both had a monodispersed spherical structure, and their average diameter was 258.1 nm. A histogram of particle size distribution is shown in Figure 3. TEM was applied to evaluate the structure and physical characteristics of the MSNs before and after pesticide loading. In Figure 2a,c, it can be seen that MSNs and Fen@MSNs have similar structures, average diameters, and dispersion, which verifies the SEM results. It can be seen in Figure 2b that there was a highly ordered mesoporous structure, one of the typical structures of MSNs, visible on the surface of the MSNs before Fenoxanil loading. The surface morphology of the Fen@MSNs showed no obvious difference from that of the MSNs (Figure 2d). The mesoporous structure could affect the uptake behaviors of the loaded compounds [28]. In order to evaluate the Fenoxanil loading potential of the MSNs, Brunauer–Emmett–Teller (BET) surface area analysis and Barrett–Joyner–Halenda (BJH) pore size and volume analysis were used to confirm the mesoporous structure of the nanoparticles. After the MSNs were loaded with Fenoxanil, the BET specific surface area decreased sharply from 1356.04 to 642.48 m^2^/g. As shown in Figure 4, the type IV isotherm curve of the MSNs increased gradually from 0 to 0.4 P/P0. Compared to MSNs, Fen@MSNs showed an obvious reduction in adsorption capacity and surface area, since most mesoporous pores were blocked by Fenoxanil. This result was confirmed by the Fourier transform infrared (FTIR) spectra (Figure 5), in which the absorption bands at 1673 cm^−1^ and 1483 cm^−1^ were attributed to characteristic peaks of Fenoxanil, and the absorption band at 1072 cm^−1^ was assigned to the MSNs. Both types of absorption bands were observed for Fen@MSNs, confirming that the MSNs were successfully loaded with Fenoxanil.

### 2.2. In Vitro Release of Fenoxanil

**Figure 6:**
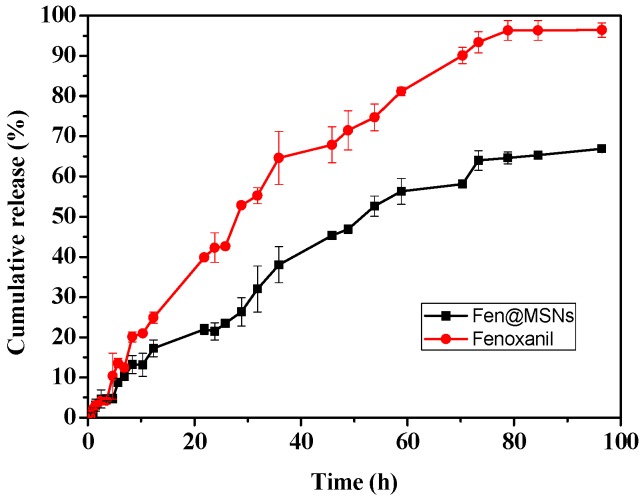
Release rates of Fenoxanil from Fen@MSNs at room temperature.

The release profiles of Fenoxanil and Fen@MSNs are shown in Figure 6. Because of the poor solubility of Fenoxanil in water, a 20% aqueous acetonitrile (ACN) solution with Tween-80 emulsifier was used as release medium. After 30 h, the amount of Fenoxanil in the release medium was greater than 50%, and its cumulative release reached nearly 100% after 100 h. On the contrary, pesticide loaded into MSNs was released slowly, with a cumulative release of about 60% after 100 h. As shown in Table 1, the cumulative release rates were fitted to three different mathematical models. Comparison of the correlation coefficients (*R*^2^) indicated that the release rates can best be described by the first order equation *y* = a*(1 − exp(−b*x)). The present study suggests that MSNs can control the release of Fenoxanil. With the sustained release of the active compound, the effective time can be prolonged to meet the requirements of disease prevention [29].

### 2.3. Analytical Method Validation

To eliminate any inaccurate results produced by matrix effects, matrix-matched calibration solutions were used to test the linearity of the method. Matrix-matched calibration solutions were obtained for leaves, roots, stem, rice, soil, and water by preparing blank extracts spiked with different concentrations of Fenoxanil in the range of 0.01–1 mg/L. The quantitative results of the analysis method were mainly dependent on linearity. As shown in Table 2, good linearity was obtained for Fenoxanil with all coefficients of determination (*R*^2^) ≥ 0.9276. In this study, the recovery amount was measured as a percentage of the amount of Fenoxanil originally spiked to the blank samples. The repeatability of the proposed method was expressed as a relative standard deviation (RSD; *n* = 5). The precision and accuracy of the method was investigated for blank leaf, stem, rice, root, soil, and water samples spiked with Fenoxanil at three different concentrations (0.01, 0.1 and 1 mg/kg). For each concentration, five spiked tests were repeated for each matrix (*n* = 5). Limits of quantification (LOQs), average recoveries, and relative standard deviations are shown in Table 3. The recoveries of Fenoxanil were in the range of 77–110% for all the samples, and RSDs were no greater than 10% for all cases. LOQs were determined as the lowest concentration of Fenoxanil detected by the instrument.

### 2.4. Translocation of Fluorescein Isothiocyanate (FITC)-Labeled MSNs in Rice Plants

**Figure 7:**
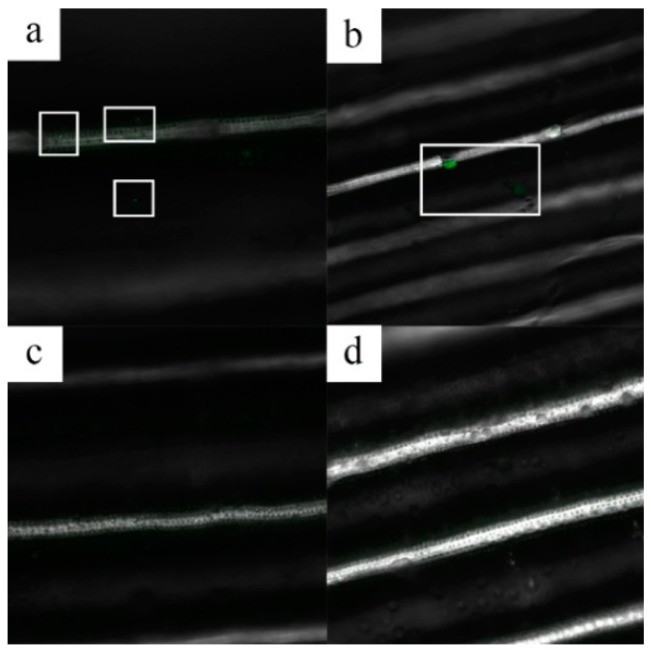
Laser scanning microscopy (LSM) images of rice plants 24 h after MSN-fluorescein isothiocyanate (FITC) treatment. (**a**) Treated plant; (**b**) Treated plant (**c**) Blank plant; (**d**) Blank plant

To study the uptake of MSNs from water, their distribution through the roots, and their delivery to the leaves, rice plants were grown in a hydroponic system. MSNs were labeled with FITC to make them visible under a laser scanning microscope (LSM). It was found that FITC-labeled MSNs could be quickly absorbed by roots from water, transported to the stems, and then transferred to the leaves. This demonstrates that, in rice plants, Fenoxanil can be carried by MSNs from the site of delivery to other tissues. These results are similar to those reported by Nair et al., who showed that FITC-labeled MSNs could be absorbed quickly by rice seedlings [30]. As seen from the LSM images of blank and treated rice samples (Figure 7), the green dot are FITC-labeled MSNs were found in rice leaves as shown in Figure 7a,b, but were not detected in blank rice plants. As it has been previously reported that MSNs can be transported in plants and can carry active compounds to different tissues [31,32] MSNs might be able to enter the plant from the surface of leaves [15], then be transported in both vessels and sieve tubes, and finally be distributed to various parts of the plant. No adverse effects were observed in the rice grown in the hydroponic system containing the suspension of FITC-labeled MSNs.

### 2.5. Uptake and Distribution of Fenoxanil-Loaded Mesoporous Silica Nanoparticles in Rice plants

**Figure 8:**
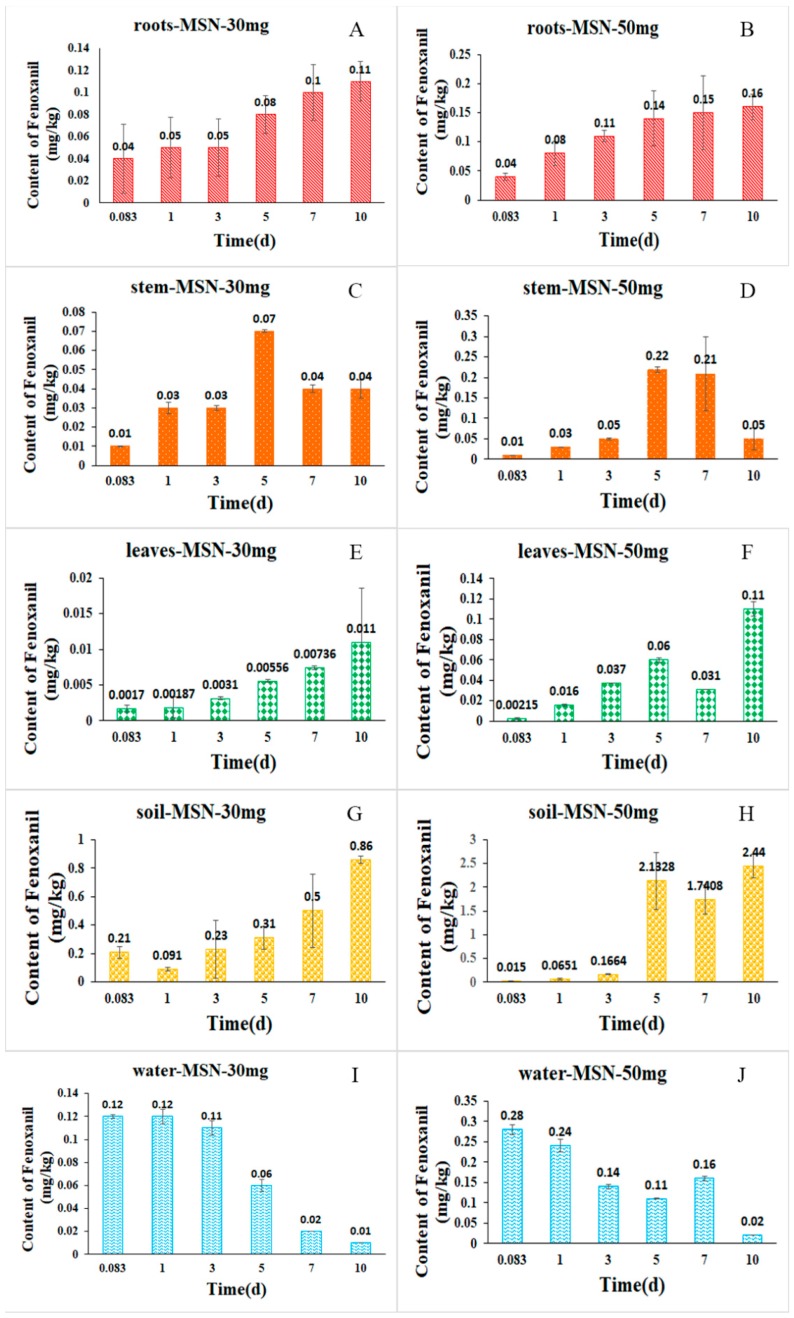
Distribution of Fennoxanil in rice plants: (**A**) roots with 30 mg/L treatment; (**B**) roots with 50 mg/L treatment; (**C**) stem with 30 mg/L treatment; (**D**) stem with 50 mg/L treatment; (**E**) leaves with 30 mg/L treatment; (**F**) leaves with 50 mg/L treatment; (**G**) soil with 30 mg/L treatment; (**H**) soil with 50 mg/L treatment; (**I**) water with 30 mg/L treatment; (**J**) water with 50 mg/L treatment.

The results of the present study demonstrated that the uptake and distribution of Fenoxanil-loaded MSNs within various tissues of rice plants and environmental substrates, i.e., the roots, stem, leaves, soil, and water, is possible. It can be seen in Figure 8a,b that the concentration of Fenoxanil in the roots increased from 0.04 to 0.11 mg/kg from 2 h to 10 days post-treatment. Similarly, in the leaves and soil, the concentration of Fenoxanil increased gradually, as shown in Figure 8e–h. On the contrary, the concentration of Fenoxanil decreased over time in the water. In the stems, the concentration of Fenoxanil increased until day five of sampling and then decreased. This shows that the uptake and retention of Fen@MSNs might vary across different rice tissues and environmental samples. In this study, the uptake and distribution behavior of Fen@MSNs in rice plants was examined at two dosages of Fenoxanil (30 and 50 mg/L). Generally, the concentrations of Fenoxanil in the samples were higher when 50 mg/L of Fenoxanil was used compared to 30 mg/L of Fenoxanil (Figure 8). However, there were no obvious differences in the distribution patterns of the pesticide between the 30 and 50 mg/L treatments, which showed that the dosage had no effect on the distribution of Fen@MSNs in rice plants.

### 2.6. Final Residues of Fenoxanil in Rice

The results showed that the final concentrations of Fenoxanil residue in rice were lower than 0.001 mg/kg. The maximum residue limit (MRL) for Fenoxanil in rice is 1 mg/kg in both China and Japan. Obviously, the Fenoxanil residues in rice were under the MRL value after the rice plants were treated by Fenoxanil-loaded MSNs. This suggests that the delivery of Fenoxanil into rice by MSNs poses only a low risk of residual Fenoxanil accumulation.

## 3. Materials and Methods

### 3.1. Materials

Fenoxanil (92.5%) was provided by Jiangsu Changqing Agrochemical Co., Ltd. CTAB (99%) was obtained from J&K Scientific Ltd. (Beijing, China). TEOS (99%) was purchased from Fluorochem Ltd. (Hadfield, UK). Deionized water was obtained from a Milli-Q water purification system from Millipore (Burlington, MA, USA). Primary secondary amine (PSA) and graphitized carbon black (GCB) were purchased from Agela Technologies (Tianjin, China). All other chemicals and reagents were commercially available and used as received without further purification. 

### 3.2. Synthesis and Characterization of Mesoporous Silica Nanoparticles

A volume of 480 mL of deionized water was used to dissolve 1.0 g of CTAB. Under constant stirring, 3.5 mL of sodium hydroxide (2.0 M) was then added to the CTAB solution at room temperature. After the temperature of the solution reached 70 °C in an oil bath, 5.0 mL of TEOS was added to the mixture in a dropwise manner at the rate of 1.0 mL/min. The solution was then stirred vigorously at 80 °C for 6 h. This process resulted in the formation of white MSNs solid particles, which were then washed three times each with ethanol and deionized water and subsequently freeze-dried under a vacuum. To remove the surfactant, the synthesized MSNs were calcined at 550 °C for 6 h. Electron microscopy studies were carried out with an SEM (SU8000; Hitachi Ltd.; Tokyo, Japan) and a TEM (JEM-200CX; Jeol Ltd., Tokyo, Japan) in order to study the structural and morphological features of Fen@MSNs and MSNs. The particle size of MSNs was analyzed by dynamic light scattering (DLS) (Malvern Z90; Malvern Instruments, Malvern, UK). The Fourier transform infrared (FT-IR) spectra of the samples were recorded with an FTIR spectrometer (NICOLET 6700; Thermo Scientific; Waltham, MA, USA) with a potassium bromide pellet. Spectra were recorded over the spectral region of 400 to 4000 cm^−1^ at a spectral resolution of 4 cm^−1^. In order to examine their pore characteristics, the nitrogen adsorption of MSNs and Fen@MSNs was studied using a surface area and pore size analyzer (TriStarII 3020; Micromeritics Instruments Corporation; Norcross, GA, USA) at 196 °C. Samples were degassed at 80 °C for 12 h prior to analysis. The mesoporous structure characteristics were analyzed using the BET and BJH procedures applied to the adsorption branches of the isotherms.

### 3.3. Loading of Fenoxanil into Mesoporous Silica Nanoparticles

Synthetic MSNs (300 mg) were added into the Fenoxanil-methanol solution (4.0 mg/mL, 11.5 mL). The mixture was ultrasonicated for 35 min, and then centrifuged at 10,000 rpm for 10 min to remove the supernatant. The Fenoxanil-loaded MSNs were dried at 50 °C for 5 h to remove the supernatant completely. The loading efficiency of Fenoxanil was measured by high-performance liquid chromatography (HPLC) using a 1200-DAD (Diode Array Detector; Agilent; Santa Clara, CA, USA). In brief, 40 mg of Fen@MSNs were dissolved in 10.0 mL of acetonitrile under sonication for 20 min, then subject to centrifugation at 10,000 rpm for 5 min, after which the supernatant was put into a volumetric flask. This process was repeated several times, and the acetonitrile solution was combined for HPLC analysis. For the HPLC analysis, a Venusil XBP-C18 column (2.5 mm × 4.6 mm, 5 µm; Bonna-Agela Technologies Inc.; Tianjin, China) was used to separate the target compound from others at 30 °C. Acetonitrile/water (70:30) was used as mobile phase at a flow rate of 1.0 mL/min. DAD signals were recorded at 230 nm. The loading efficiency (%) was calculated as (weight of pesticide in nanoparticles/weight of nanoparticles) × 100%. In this study, the loading efficiency of MSNs was 14.8%.

### 3.4. *Synthesis of FITC-labeled MSNs*

To make the MSNs visible in rice plants, they were labeled with FITC. A total of 500 mg of MSNs were added to 40 mL of methylbenzene and, after ultrasonic dispersion for 15 min, the suspension was heated to 80 °C under vigorous stirring. Later, 100 μL of (3-Aminopropyl)triethoxysilane (APTES) was introduced into the suspension and the sample was prepared by refluxing at 110 °C. To remove excess APTES, the MSNs were washed three times with water and ethanol. Subsequently, 150 mg of FITC was dissolved in 150 mL of ethanol. The mixture was magnetically stirred vigorously for 24 h at room temperature to cross connect the APTES and the FITC. The FITC-labeled MSNs were washed three times with ethanol and deionized water to remove excess FITC, centrifuged at 10,000 rpm for 3 min, and then freeze-dried. To prevent the fluorescence intensity from decreasing, the prepared nanoparticles were covered with aluminum foil and stored at 4 °C.

### 3.5. Fenoxanil Release

Fen@MSNs (20 mg) were dispersed in 250 mL of phosphate buffer (pH = 6.93) containing 0.1% Tween-80 emulsifier, which was used as the release medium. A D-800LS dissolution tester (Tianjin University, Tianjin, China) was used for the release test at a stirring speed of 120 rpm. In order to verify the sustained release property of Fen@MSNs, the cumulative release rate of Fenoxanil from the nanoparticles was calculated by measuring its concentration in the release medium. For HPLC analysis, 0.8 mL of the release medium was withdrawn from the assay at given time intervals. It was replaced with an equal volume of Tween-80 containing phosphate buffer, in order to maintain a constant volume of assay solution. The release test was performed duplicates. The accumulative release can be calculated by the following equation:

$$E_{r}=\frac{Ve\sum_{1}^{n-1}C_{i}+V_{0}C_{n}}{M_{p}}\times 10$$

*E_r_*: the accumulative release (%) of Fenoxanil from the nanoparticles;
*Ve*: the volume of the release medium taken in a time interval (*Ve* = 0.8 mL);
*C_i_*: the Fenoxanil concentration in the release medium;
*i*: the release time
*V*_0_: the volume of the release medium (250 mL);
*n*: the sample number;
*M_p_*: the total amount of pesticide entrapped in the nanoparticles.

### 3.6. Greenhouse Study

Rice plants were cultivated in pools made of concrete and potting soil. The Fen@MSNs were weighed and added into the pool at the heading period of rice growth. Two concentration levels of Fenoxanil were used for the uptake and distribution study: 30 mg/L and 50 mg/L (The different amounts of Fen@MSNs corresponding to both 30 mg/L and 50 mg/L total Fenoxanil.). In order to characterize the uptake and distribution behavior of Fenoxanil in rice plants during different growth durations, representative samples were collected 2 h, 1, 3, 5, 7, and 10 days after the application of Fen@MSNs. Each treatment was repeated three times. The Fenoxanil concentration in the roots, leaves, and stems of the rice plants, as well as in the paddy water and paddy soil were determined over a period of 10 days using high-performance liquid chromatography–tandem mass spectrometry (HPLC–MS/MS). The concentration levels of Fenoxanil in rice were also determined during the harvest time.

### 3.7. Sample Preparation

Homogenized sample aliquots (2 ± 0.1 g) were weighed and each was added to a 50-mL polypropylene screw-cap centrifuge tube. For validation of the recovery studies, negative controls were prepared by adding an appropriate volume of working standard solution to blank samples. The tubes containing the target samples were incubated for 2 h at room temperature to distribute the pesticide evenly. Ultrapure water (5 mL) and acetonitrile (10 mL) were then added to these samples, and the tubes were shaken vigorously at an oscillation frequency of 1350 min^−1^ for 10 min using a CK-2000 high-throughput grinder (TH Morgan, Beijing, China). Subsequently, 1 g of NaCl and 4 g of anhydrous MgSO4 were added. The tubes were immediately shaken again for 5 min and then centrifuged for 5 min at a relative centrifugal force (RCF) of 2811× *g* using a SIGMA 3-15 centrifuge (SIGMA, Taufkirchen, Germany). After extraction, 1.5 mL of the supernatant was transferred into a single-use centrifuge tube containing 20 mg of GCB and 30 mg of PSA. The samples were then vortexed for 1 min and centrifuged for 5 min at an RCF of 2400× *g*. The upper layer was filtered through a 0.22-µm nylon syringe filter into an auto-sampler vial for HPLC–MS/MS injection. Matrix-matched standard solutions were used to eliminate any possible matrix effects. The retention time of Fenoxanil was 5.05 min. Two ion transitions were chosen: *m*/*z* 328→189 (quantification), and *m*/*z* 328→302 (confirmation). ACN and formic acid water (*v*/*v* = 0.1%) were chosen for the mobile phase, the flow gradients were: 35% ACN and 65% water at 0 min; 85% ACN and 15% water at 1.8 min; 35% ACN and 65% water at 6.5 min; and 35% ACN and 65% water at 7 min.

## 4. Conclusions

Numerous studies have shown that MSNs can be used as carriers to deliver pesticides into plants. This is considered to be an effective method of reducing the amount of pesticide used. However, this new method raises significant concerns about its behavior in plants. The uptake and distribution behavior of nanoparticles in plants needs to be uncovered before the method can be widely applied in the field. In this study, Fen@MSNs were synthesized and used on rice. It was shown that Fen@MSNs can be delivered to and absorbed by rice plants, and the dosage has almost no effect on the distribution of Fen@MSNs in rice plants. The results also suggest that the distribution behavior of pesticides in plants could be regulated by MSNs. The application of Fen@MSNs in water carries only a low risk of Fenoxanil accumulating in the edible part of the plant. This work can help us understand the effects of nanopesticides when used on plants. It provides insight into the fate of Fen@MSNs in different samples in hydroponic system.

## Figures and Tables

**Table 1:** Mathematical models of Fenoxanil release from Fenoxanil and Fen@MSN solutions.

Sample	Models	Equation	Fitting Equation	*R* ^2^
Fenoxanil	Zero-order equation	*y* = a + b*x*	4.266 + 1.42*x*	0.95371
	First-order equation	*y* = a*(1 − exp(−b*x))	126.56*(1 − exp(−0.016*x))	0.99416
	Higuchi	*y* = a*(*x*^(1/2)) + b	10.725*x*^1/2^ + 0.28305	0.98287
Fen@MSNs	Zero-order equation	*y* = a + b*x*	3.3376 + 0.77*x*	0.98111
	First-order equation	*y* = a*(1 − exp(−b*x))	89.19*(1 − exp(−0.015*x))	0.99764
	Higuchi	*y* = a*(*x*^(1/2)) + b	7.654*x*^1/2^ − 6.6	0.99054

**Table 2:** The standard calibration curve (*R*^2^), limit of detection (LOD), and limit of quantification (LOQ) of Fenoxanil in different matrices.

Compound	Matrix	Standard Calibration Curve		LOD (mg/kg)	LOQ (mg/kg)
		Regression Equation	*R* ^2^		
Fenoxanil	roots	*y* = 588191*x* + 14086	0.9968	0.0001	0.001
	stem	*y* = 536119*x* + 11018	0.9997	0.0001	0.001
	rice	*y* = 513143*x* + 11661	0.9938	0.0001	0.001
	leaves	*y* = 383365*x* + 15639	0.97	0.0001	0.001
	soil	*y* = 565884*x* + 12994	0.9985	0.0001	0.001
	water	*y* = 529117*x* + 33323	0.9276	0.0001	0.001

**Table 3:** Accuracy and precision of the analysis method in different matrices at three levels of spiked Fenoxanil.

Sample	Spiked Level (mg/kg)	Average Recoveries (%)	RSD (%)	LOQ (mg/kg)
Roots	1.0	85	3	0.001
	0.1	101	6	
	0.01	89	4	
Leaves	1.0	79	2	0.001
	0.1	95	6	
	0.01	88	7	
Stems	1.0	87	9	0.001
	0.1	90	3	
	0.01	105	6	
Rice	1.0	88	8	0.001
	0.1	79	4	
	0.01	99	7	
Water	1.0	92	7	0.001
	0.1	78	9	
	0.01	102	8	
Soil	1.0	91	4	0.001
	0.1	77	5	
	0.01	110	8	

## Abbreviations

MSNs	mesoporous silica nanoparticles
Fen@MSNs	Fenoxanil-loaded MSNs
CTAB	cetyltrimethylammonium bromide
PSA	primary secondary amine
GCB	graphitized carbon black
FITC	fluorescein isothiocyanate
APTES	(3-Aminopropyl)triethoxysilane
SEM	scanning electron microscope
TEM	transmission electron microscope
BET	Brunauer–Emmett–Teller
BJH	Barrett–Joyner–Halenda
DLS	dynamic light scattering
FTIR	Fourier transform infrared
DAD	diode array detector
HPLC	high-performance liquid chromatography
HPLC–MS/MS	high-performance liquid chromatography–tandem mass spectrometry
RSD	relative standard deviation
LOD	limit of detection
LOQ	limit of quantification
LSM	laser scanning microscope
MRL	maximum residue limit
